# Combination of *isocitrate dehydrogenase 1 (IDH1*) mutation and podoplanin expression in brain tumors identifies patients at high or low risk of venous thromboembolism

**DOI:** 10.1111/jth.14129

**Published:** 2018-05-20

**Authors:** P. Mir Seyed Nazari, J. Riedl, M. Preusser, F. Posch, J. Thaler, C. Marosi, P. Birner, G. Ricken, J. A. Hainfellner, I. Pabinger, C. Ay

**Affiliations:** ^1^ Clinical Division of Hematology and Hemostaseology Department of Medicine I Comprehensive Cancer Center Center Medical University of Vienna Vienna Austria; ^2^ Clinical Division of Oncology Department of Medicine I Comprehensive Cancer Center Medical University of Vienna Vienna Austria; ^3^ Division of Oncology Department of Internal Medicine Medical University of Graz Graz Austria; ^4^ Clinical Institute of Pathology Medical University of Vienna Vienna Austria; ^5^ Institute of Neurology Medical University of Vienna Vienna Austria

**Keywords:** brain neoplasms, cancer, glioma, thromboembolism, thrombosis

## Abstract

**Essentials**

- Risk stratification for venous thromboembolism (VTE) in patients with brain tumors is challenging.
- Patients with *IDH1* wildtype and high podoplanin expression have a 6‐month VTE risk of 18.2%.
- Patients with *IDH1* mutation and no podoplanin expression have a 6‐month VTE risk of 0%.
- *IDH1* mutation and podoplanin overexpression in primary brain tumors appear to be exclusive.

**Summary:**

**Background**

Venous thromboembolism (VTE) is a frequent complication in primary brain tumor patients. Independent studies revealed that podoplanin expression in brain tumors is associated with increased VTE risk, whereas the *isocitrate dehydrogenase 1 (IDH1)* mutation is associated with very low VTE risk.

**Objectives**

To investigate the interrelation between intratumoral podoplanin expression and *IDH1* mutation, and their mutual impact on VTE development.

**Patients/Methods**

In a prospective cohort study, intratumoral IDH1 R132H mutation and podoplanin were determined in brain tumor specimens (mainly glioma) by immunohistochemistry. The primary endpoint of the study was symptomatic VTE during a 2‐year follow‐up.

**Results**

All brain tumors that expressed podoplanin to a medium‐high extent showed also an *IDH1* wild‐type status. A score based on *IDH1* status and podoplanin expression levels allowed prediction of the risk of VTE. Patients with wild‐type *IDH1* brain tumors and high podoplanin expression had a significantly increased VTE risk compared with those with mutant *IDH1* tumors and no podoplanin expression (6‐month risk 18.2% vs. 0%).

**Conclusions**

*IDH1* mutation and podoplanin overexpression seem to be exclusive. Although brain tumor patients with *IDH1* mutation are at very low risk of VTE, the risk of VTE in patients with *IDH1* wild‐type tumors is strongly linked to podoplanin expression levels.

## Introduction

Venous thromboembolism (VTE) is a frequent complication in brain tumor patients 1. Recently, novel insights into the pathophysiology of brain tumor‐associated VTE have been generated. We reported in a previous study that the expression of podoplanin by tumor cells led to platelet aggregation and was associated with increased risk of VTE 2. Podoplanin is a sialomucin‐like glycoprotein and has the ability to induce platelet aggregation via interacting with the C‐type lectin‐like receptor (CLEC)‐2 on platelets 3. Recent experimental studies using mouse models confirmed the important role of podoplanin in the pathophysiology of VTE 4, 5, 6. Based on these observations, podoplanin‐induced platelet aggregation might be a major driver of brain tumor‐associated VTE. Moreover, a study by Unruh *et al*. reported that a subgroup of glioma patients with tumors harboring the *isocitrate dehydrogase 1 (IDH1)* mutation, which leads to the production of D‐2‐hydroxyglutarate (D‐2‐HG), are at very low risk of VTE 7. The authors suggested that the risk of VTE in *IDH1* mutant glioma patients is reduced by the inhibitory effect of D‐2‐HG on platelet aggregation. Interestingly, D‐2‐HG is an oncometabolite that is associated with DNA hypermethylation of several genes, including coagulation‐associated genes such as tissue factor (TF) 7 8.

Currently, risk stratification for VTE in patients with primary brain tumors still remains challenging. However, the interrelation between podoplanin and *IDH1* mutation and their combined effect on risk of VTE has not been explored up to date. Here, we evaluated both molecular markers in a clinical prospective cohort study of patients with primary brain tumors and their association with prediction and risk stratification of VTE.

## Methods

### Study design and population

The study was performed within the framework of the Vienna Cancer and Thrombosis Study (CATS), a prospective observational cohort study. The aim of CATS is to identify risk factors for VTE in cancer patients. The detailed study design and methods have been published previously 9, 10. Briefly, patients with newly diagnosed cancer or progressive disease after cancer remission are enrolled and followed for a period of 2 years. The primary endpoint of CATS is defined as occurrence of symptomatic and objectively diagnosed VTE. The secondary endpoint is defined as death from any cause.

### Immunohistochemistry

IDH1 R132H mutation in formalin‐fixed and paraffin‐embedded (FFPE) brain tumor samples was assessed by immunohistochemistry (IHC) via the monoclonal anti‐IDH1 R132H antibody (Dianova, Hamburg, Germany). Data on intratumoral podoplanin were available from our previous study 2. The intensity of podoplanin was semi‐quantitatively classified into four degrees as follows: negative (−); low expression (+), 50% of tumor cells express podoplanin and/or mild staining intensity; moderate expression (++), 50% to 70% of tumor cells express podoplanin at a moderate to strong intensity; and high expression (+++), 70% of tumor cells express podoplanin at a strong intensity level.

### Statistical and TCGA analysis

All statistical analyses were performed using Stata 14.0 (Stata Corp., Houston, TX, USA). Continuous variables are summarized as medians (25th–75th percentile) and categorical variables are reported as absolute counts (%). The association between variables was assessed with rank‐sum tests and χ^2^ tests, and the correlation between continuous variables using Spearman's rank‐based correlation coefficient. The cumulative incidence of VTE was estimated with competing risk cumulative incidence estimators, treating death from any cause other than fatal VTE as the competing event of interest. VTE incidences between groups were compared with log‐rank tests. Cause‐specific hazards of VTE in relation to predictor variables were modelled with univariable and multivariable Cox proportional hazards models, yielding hazard ratios (HRs) with 95% confidence intervals. Two datasets with available *IDH1* (R132H) mutation data (from whole exome sequencing), podoplanin methylation levels (from HM450 array, expressed as beta‐values) and mRNA expression levels of podoplanin (from RNA‐Seq V2, expressed on a log2‐scale) from patients with lower‐grade glioma (LGG ‘TCGA, provisional’, *n* = 280) and glioblastoma (GBM ‘TCGA, provisional’, *n* = 135) were extracted from The Cancer Genome Atlas (TCGA) 11.

## Results and discussion

### Baseline analysis

In total, 213 patients with primary brain tumors, mostly glioma, were included (Table 1). The exact tumor subtypes have been reported in our previous publication 2. Patients with podoplanin‐positive tumors (IHC score: +, ++, and +++; *n* = 151, 71%) were older, had a higher probability of having glioblastoma, and had lower platelet counts and higher D‐dimer (all *P* ≤ 0.001). An *IDH1* mutation was found in 42 tumor specimens (20%). On average, patients with *IDH1* mutant tumors were younger, suffered from diffuse or anaplastic glioma or from secondary glioblastoma, and had higher platelet counts and lower D‐dimer.

**Table 1:** Baseline characteristics including podoplanin expression, *IDH1* mutation status and peripheral blood parameters in our patient cohort

Demographics	Patients studied *n* (% miss.)		Podoplanin +, ++ and +++ (*n* = 151)	Podoplanin – (*n* = 62)	*P*‐value	*IDH1* mutant (*n* = 42)	*IDH1* wild‐type (*n* = 171)	*P*‐value
Median age at study entry	213 (0%)	55 [44–66]	60 [48–68]	46 [37–56]	<0.0001	42 [36–50]	60 [48–67]	<0.0001
Female sex	213 (0%)	79 (37%)	54 (36%)	25 (40%)	0.53	17 (40%)	62 (36%)	0.61
Body mass index (BMI, kg/m²)	213 (0%)	25 [23–28]	26 [23–28]	25 [23–27]	0.53	24 [23–27]	26 [23–28]	0.25
Newly‐diagnosed malignancy	213 (0%)	185 (87%)	136 (90%)	49 (79%)	0.03	35 (83%)	150 (88%)	0.45
Brain tumor types	213 (0%)	/	/	/	<0.0001	/	/	<0.0001
Glioblastoma/sarcoma (WHO IV)	/	152 (71%)	124 (82%)	28 (18%)	/	18 (12%)	134 (88%)	/
Anaplastic glioma (WHO III)	/	38 (18%)	14 (37%)	24 (63%)	/	18 (47%)	20 (53%)	/
Low‐grade glioma (WHO II)	/	10 (5%)	3 (30%)	7 (70%)	/	4 (40%)	6 (60%)	/
Other		13 (6%)	10 (77%)	3 (23%)	/	2 (15%)	11 (85%)	/
Laboratory parameters								
Platelet count (G L^−1^)	213 (0%)	240 [194–311]	227 [186–285]	286 [241–355]	<0.0001	297 [244–338]	232 [190–289]	0.0004
D‐dimer μg mL^−1^	190 (11%)	0.7 [0.3–1.4]	0.8 [0.5–1.9]	0.4 [0.2–0.8]	<0.0001	0.4 [0.3–0.8]	0.7 [0.4–1.7]	0.008
Soluble P‐selectin, ng mL^−1^	197 (8%)	37 [29–49]	37 [29–48]	39 [29–51]	0.51	37 [29–49]	38 [29–50]	0.91
Peak thrombin generation, nm	212 (0%)	347 [184–543]	374 [198–552]	284 [167–493]	0.12	280 [163–490]	363 [190–551]	0.23
Serum calcium (total), mmol L^−1^	124 (42%)	2.37 [2.27–2.46]	2.36 [2.25–2.44]	2.37 [2.31–2.50]	0.05	2.40 [2.31–2.50]	2.36 [2.26–2.44]	0.09
Serum calcium (albumin corrected), mmol L^−1^	124 (42%)	2.31 [2.27–2.38]	2.32 [2.27–2.39]	2.30 [2.23–2.36]	0.15	2.28 [2.22–2.37]	2.32 [2.27–2.39]	0.06

Median levels [25th–75th percentile] are given for continuous data and absolute frequencies (%) for count data. *P*‐values for the comparison between podoplanin‐positive and podoplanin‐negative tumors, as well as between *IDH1* wild‐type and *IDH1* mutation, are reported.

### *IDH1* mutation and podoplanin are inversely correlated in primary brain tumors

**Figure 1:**
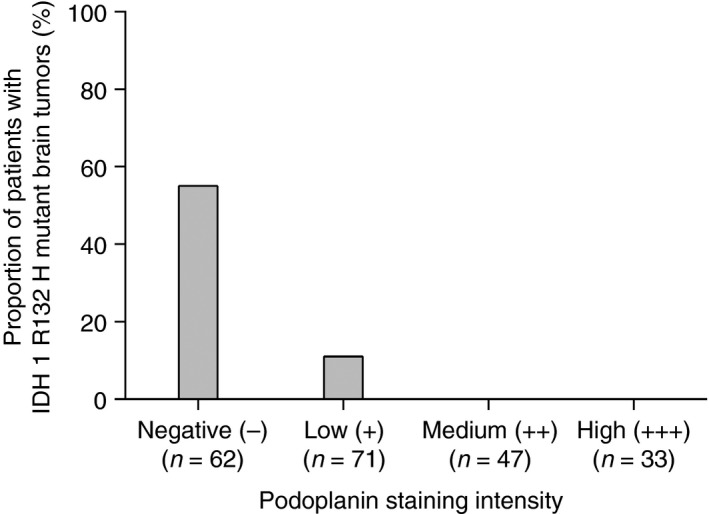
Proportion of primary brain tumors with IDH1 mutation and different levels of podoplanin expression. IDH1 R132H mutation is only found in tumors with no or low podoplanin expression.

Immunohistochemical analysis revealed a strong inverse association between podoplanin expression levels and *IDH1* mutation (*P* < 0.0001), with 34 (55%) of the 62 podoplanin‐negative tumors, but only eight (5%) of the 151 podoplanin‐positive tumors, having an *IDH1* mutation (Fig. 1). To externally validate these findings, data from the TCGA were analyzed. Here, mean podoplanin mRNA levels were 6‐fold lower in the 194 LGG patients with an *IDH1* mutation than in the 86 LGG patients with an *IDH1* wild‐type tumor (log2‐ratios: 7.1 vs. 9.6, *P* < 0.0001), and 8‐fold lower in the six GBM patients with an *IDH1* mutation than in the 129 GBM patients with an *IDH1* wild‐type tumor (log2‐ratios: 9.1 vs. 12.1, *P* = 0.0002), respectively. These results are also in concordance with previous findings by Birner *et al*., suggesting an inverse relationship of *IDH1* mutation with podoplanin expression in glioma patients 12.

To explore whether decreased podoplanin expression in *IDH1* mutant tumors may be a result of the impact of mutant *IDH1*‐dependent genome hypermethylation, we further compared podoplanin methylation between *IDH1* mutant and wild‐type tumors in the TCGA subset with available HM450 methylation data (*n* = 321). Indeed, podoplanin methylation was strongly increased in the *IDH1* mutant compared with *IDH1* wild‐type tumors, and this applied to both the LGG cohort (median beta‐value for *IDH1* mutant vs. wild‐type: 0.48 vs. 0.05, *P* < 0.0001) and the GBM cohort (0.52 vs. 0.03, *P* = 0.03). However, higher podoplanin methylation resulted in lower podoplanin mRNA expression only in LGG patients (Spearman's rho = −0.64, *P* < 0.0001) but not GBM patients (rho = −0.15, *P* = 0.35). Interestingly, Noushmehr *et al*. demonstrated that *IDH1* mutation is tightly associated with a glioma‐CpG island methylator phenotype (G‐CIMP), and podoplanin is one of the top‐ranked hypermethylated genes in G‐CIMP‐positive tumors 13.

Previously, it was reported that intravascular thrombosis occurs more frequently in glioblastoma than in other brain tumors, pointing to an intrinsic coagulant phenotype 14. However, four molecular subgroups in glioblastoma have been recently described that also differ in expression levels of coagulation‐associated genes (e.g. TF) that might be regulated by oncogenes 15, 16, 17. The so‐called ‘proneural’ subtype is highly associated with *IDH1* mutation, *platelet‐derived growth factor receptor A (PDGFRA)* alterations and very low TF expression 15, 16. In contrast, the ‘mesenchymal’ subtype is linked to higher TF and podoplanin expression as well as to *neurofibromatosis type 1 (NF1)* mutation and loss of the *phosphatase and tensin homolog (PTEN)* 15, 16, 18. Of note, PTEN is an inhibitor of the phosphatidylinositol 3‐kinase (PI3K)/Akt signaling pathway, which is also involved in the upregulation of TF and podoplanin 19, 20, 21. However, in previous studies, we demonstrated that podoplanin expression was associated with occurrence of VTE in patients with primary brain tumors and no association between TF expression and VTE risk could be found 2, 22.

### *IDH1* mutation and VTE risk in primary brain tumor patients

During a median follow‐up time of 731 days (range, 3–731 days), VTE occurred in 29 (14%) brain tumor patients. The cumulative 6‐, 12‐ and 24‐month risks of VTE were 10.1%, 13.3% and 14.8%, respectively. The presence of an *IDH1* mutation was associated with a lower risk of VTE. Only one out of 42 brain tumor patients with an *IDH1* mutation developed VTE during follow‐up in our study, and this patient had several VTE events in the medical history (already years before cancer diagnosis), suggesting that in this patient other thrombotic risk factors existed independently from the brain tumor. The cumulative 6‐, 12‐ and 24‐month VTE risks were 13.0%, 15.4% and 17.0% in patients with *IDH1* wild‐type tumors, and 0%, 2.4% and 2.4% in patients with *IDH1* mutant tumors, respectively (log‐rank *P* = 0.008). In univariable time‐to‐VTE regression, the *IDH1* mutation predicted a lower risk of VTE (hazard ratio [HR] = 0.11; 95% CI, 0.01–0.80; *P* = 0.029), and this prevailed after multivariable adjustment for glioblastoma and age (Models #1 and #2, Table 2). Thus, these clinical data externally validate previous findings demonstrating that mutant *IDH1* is a predictor of low VTE risk in patients with primary brain tumors 7. Based on a mouse model, Unruh *et al*. proposed that *IDH1* mutation leads to decreased serum calcium levels with subsequent inhibition of platelet activation, thereby preventing VTE 7. However, we were not able to confirm a statistically significant association between *IDH1* status and serum calcium concentrations in primary brain tumor patients (Table 1).

**Table 2:** Univariable and multivariable hazard ratios (HRs) for venous thromboembolism (VTE) and death in primary brain tumors patients based on *IDH1* mutation and podoplanin

Outcome	Model	Variable	HR	95% CI	*P*
VTE	Model #1	IDH1 R132H mutation (*n* = 42)	0.11	0.01–0.80	0.029
	Model #2	IDH1 R132H mutation (*n* = 42)	0.11	0.01–0.83	0.032
		Glioblastoma (*n* = 152)	2.31	0.79–6.76	0.127
		Age (per 10 years increase)	0.83	0.62–1.12	0.222
	Model #3*	0 points (*n* = 34)	Ref.	Ref.	Ref.
		1 point (*n* = 36)	3.13	0.33–30.10	0.323
		2 points (*n* = 63)	6.65	0.84–52.64	0.073
		3 points (*n* = 47)	8.40	1.04–67.58	0.045
		4 points (*n* = 33)	13.28	1.65–106.97	0.015
Death	Model #4	IDH1 R132H mutation (*n* = 42)	0.19	0.09–0.39	<0.0001
	Model #5	IDH1 R132H mutation (*n* = 42)	0.30	0.14–0.64	0.002
		Glioblastoma (*n* = 152)	2.07	1.18–3.63	0.011
		Age (per 10 years increase)	1.26	1.08–1.48	0.004
	Model #6*	0 points (*n* = 34)	Ref.	Ref.	Ref.
		1 point (*n* = 36)	1.93	0.76–4.90	0.168
		2 points (*n* = 63)	4.89	2.18–10.96	<0.0001
		3 points (*n* = 47)	7.21	3.17–16.40	<0.0001
		4 points (*n* = 33)	6.79	2.89–15.97	<0.0001

CI, confidence interval. *The score is based on the sum of *IDH1* status (mutant = 0, wild‐type = 1) and podoplanin expression levels (− = 0, + = 1, ++ = 2 and +++ = 3).

### *IDH1* and podoplanin are joint prognostic markers for brain tumor‐associated VTE

**Figure 2:**
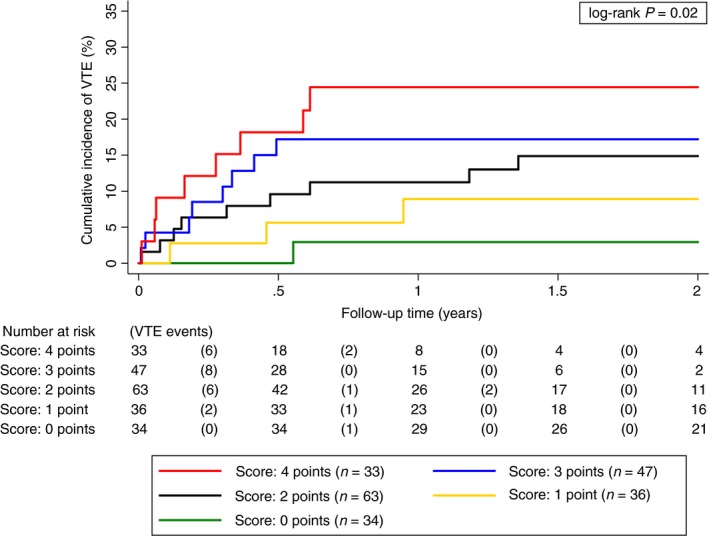
Cumulative incidence of venous thromboembolism accounting for competing mortality according to the IDH1 status and podoplanin expression in primary brain tumor patients. To predict the risk of brain cancer‐associated VTE, a score was established based on the sum of the IDH1 status (mut = 0, wt = 1) and podoplanin expression levels (no = 0, low = 1, medium = 2, high = 3). Brain tumor patients with the combination of IDH1wt and high podoplanin expression were at highest risk of VTE, whereas patients with IDH1 R132H mutation combined with no podoplanin expression showed the lowest risk of developing VTE during the follow‐up time. As IDH1 mutation and podoplanin overexpression (medium, high) appear to be exclusive, only the score of 1 included a heterogenous subgroup of tumors. All other scores (0, 2–4) included homogenous subgroups regarding IDH1 status and podoplanin expression levels: score 0 = IDH1mut with no podoplanin; score 1 = IDH1mut with low podoplanin and IDH1wt with no podoplanin; score 2 = IDH1wt with low podoplanin; score 3 = IDH1wt with medium podoplanin; score 4 = IDH1wt with high podoplanin (mut, mutant; wt, wild‐type). [Colour figure can be viewed at http://wileyonlinelibrary.com]

To quantify a potential additive contribution of *IDH1* and podoplanin towards the risk of VTE, we evaluated the sum of *IDH1* status (mutant = 0, wild‐type = 1) and podoplanin staining intensity (− = 0, + = 1, ++ = 2 and +++ = 3) as a putative predictor of brain tumor‐associated VTE. The 6‐month risk of VTE was 0%, 5.6%, 9.6%, 17.2% and 18.2% in patients with 0 (*n* = 34), 1 (*n* = 36), 2 (*n* = 63), 3 (*n* = 47) and 4 (*n* = 33) points of this joint *IDH1* and podoplanin sum, respectively (log‐rank *P* = 0.02, Fig. 2). Brain tumors with both wild‐type *IDH1* and high podoplanin expression were associated with increased risk of VTE compared to those with mutant *IDH1* and no podoplanin expression (follow‐up time of 2 years: HR = 13.28, 95% CI 1.65–106.97, *P* = 0.015) (Table 2). Based on our results, the risk of developing VTE in patients with wild‐type *IDH1* is strongly linked to intratumoral podoplanin expression levels. Thus, the combination of *IDH1* mutation and podoplanin expression might be a useful immunohistochemical marker for VTE risk assessment in patients with primary brain tumors. In addition, *IDH1* and podoplanin are also joint prognostic markers for overall survival in patients with primary brain tumors (Table 2).

Mechanistically, we suggest that podoplanin induces platelet aggregation via the platelet receptor CLEC‐2, which might subsequently trigger systemic thrombosis. Recent studies have shown that thrombosis is induced by podoplanin upregulation and its interaction with CLEC‐2: Shirai *et al*. demonstrated that CLEC‐2‐deficiency in mice was protective against thrombosis and hematogenous metastasis. Thrombus formation in tumor vessels was significantly inhibited in CLEC‐2‐depleted mice bearing podoplanin‐expressing B16F10 melanoma cells 4. Payne *et al*. again revealed that mice deficient in CLEC‐2 were protected against deep vein thrombosis (DVT) 6. Furthermore, they demonstrated that podoplanin was highly expressed in the inferior vena cava wall of wild‐type mice that developed thrombosis and inhibition of podoplanin reduced the size of the thrombus.

Our study has some limitations. Intratumoral podoplanin levels were determined only semi‐quantitatively. In practice, this might lead to subjective and laboratory‐specific variability. To address this issue, all tumor sections were reviewed by two experienced neuropathologists and no disagreement was observed. Moreover, *IDH1* mutation was assessed immunohistochemically rather than on a genetic level in our clinical study. Finally, we were not able to provide *in vivo* evidence confirming the mechanistic pathways leading to thrombosis. However, we extracted mRNA and methylation data from a public database (TCGA) to look closer at the interrelation between the *IDH1* mutation status and podoplanin. *IDH1* mutation was associated with podoplanin hypermethylation, presumably via D‐2‐HG, and inversely linked to podoplanin overexpression. As discussed, we assume that in *IDH1* wild‐type tumors the amount of podoplanin upregulation might depend on *PTEN* loss and PI3K/Akt signaling 15, 18, 19.

In summary, primary brain tumors, which harbor the *IDH1* mutation, are associated with a very low risk of VTE, whereas a high risk of VTE is observed in patients with *IDH1* wild‐type tumors*. IDH1* wild‐type was associated with upregulation of podoplanin, and increasing podoplanin expression levels were associated with a gradual increase in the risk of VTE. We concluded that measurement of *IDH1* mutation and podoplanin allows differentiating between subgroups of brain tumors that show distinct VTE risk profiles. Whether primary thromboprophylaxis in high‐risk patients based on IHC staining for *IDH1* mutation and podoplanin expression is able to reduce the risk of VTE needs to be investigated in clinical trials. Our proposed combination marker panel of *IDH1* mutation and podoplanin could be used to enrich these trials with patients who have the highest risk of VTE and might benefit most from primary thromboprophylaxis compared with the risk of intracerebral bleeding.

## Addendum

P. M. S. Nazari, J. Riedl, I. Pabinger and C. Ay designed the study; P. M. S. Nazari, J. Riedl, M. Preusser, P. Birner, G. Ricken and J. A. Hainfellner designed and performed the experiments; J. Riedl, M. Preusser, C. Marosi, J. Thaler and C. Ay recruited patients; P. M. S. Nazari, J. Riedl, C. Ay and F. Posch performed statistical analyses; and P. M. S. Nazari, J. Riedl and C. Ay wrote the article, which was reviewed and edited by all the other authors.

## Disclosure of Conflict of Interests

The authors state that they have no conflict of interest.
